# Force fluctuations in three-dimensional suspended fibroblasts

**DOI:** 10.1098/rstb.2014.0028

**Published:** 2015-02-05

**Authors:** Florian Schlosser, Florian Rehfeldt, Christoph F. Schmidt

**Affiliations:** Third Institute of Physics—Biophysics, Georg August University, 37077 Göttingen, Germany

**Keywords:** cell mechanics, optical trap, cell cortex, non-muscle myosin II, active matter

## Abstract

Cells are sensitive to mechanical cues from their environment and at the same time generate and transmit forces to their surroundings. To test quantitatively forces generated by cells not attached to a substrate, we used a dual optical trap to suspend 3T3 fibroblasts between two fibronectin-coated beads. In this simple geometry, we measured both the cells' elastic properties and the force fluctuations they generate with high bandwidth. Cell stiffness decreased substantially with both myosin inhibition by blebbistatin and serum-starvation, but not with microtubule depolymerization by nocodazole. We show that cortical forces generated by non-muscle myosin II deform the cell from its rounded shape in the frequency regime from 0.1 to 10 Hz. The amplitudes of these forces were strongly reduced by blebbistatin and serum starvation, but were unaffected by depolymerization of microtubules. Force fluctuations show a spectrum that is characteristic for an elastic network activated by random sustained stresses with abrupt transitions.

## 1. Introduction

Many cellular processes such as substrate– or cell–cell adhesion, locomotion or cell division [1–6] depend critically on mechanical interactions and the forces cells exert and experience. Cells generate forces and use them to move through tissues, but they also derive signals about their environment from forces. Much research has been performed on the dependence of shape and internal structure of cultured cells on substrate stiffness [7–9]. The most prominent structures that develop in the acto-myosin cell cortex are contractile stress fibres that can be highly ordered [9].

Cellular forces are generated mainly by myosin motors interacting with actin filaments and hydrolysing ATP [10]. Forces are also generated by non-equilibrium polymerization and depolymerization of microtubules and actin filaments [11]. The dynamics of such ‘active matter’ is determined by the interplay between the microscopic force generation and the viscoelastic properties of the material. Mechanical properties and non-equilibrium fluctuations of such systems have been successfully studied in reconstituted *in vitro* model systems [12,13], including networks encapsulated in a lipid envelope mimicking cellular geometry [14]. However, the accurate measurement of mechanical properties of living cells and of the forces they generate remains challenging. Most work in this direction has been done on substrate-adherent cultured cells [15], a rather non-physiological situation for most cell types.

**Figure 1.**
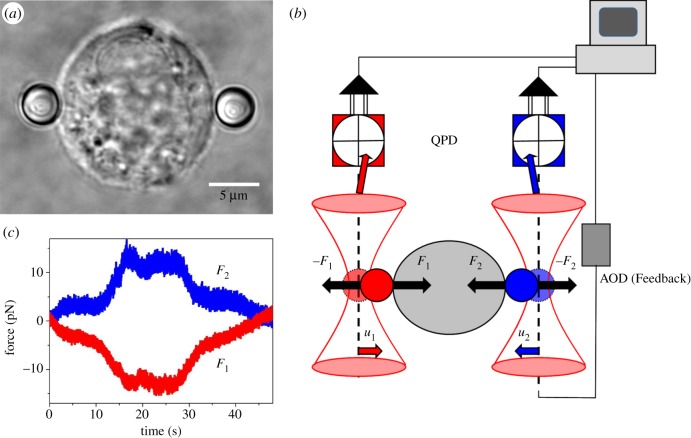
Cell suspended between two optical traps. (*a*) 3T3 fibroblast cell suspended between two optically trapped 4 µm fibronectin-coated polystyrene beads. (*b*) Schematic of the set-up: a cell (grey) exerts forces *F*_1_ and *F*_2_ on the two attached beads (red and blue). The corresponding displacements of the beads (*u*_1_ and *u*_2_) in the traps are detected by QPDs and recorded via an FPGA I/O board and LabView software. In feedback mode, the computer controls the AOD and displaces one of the traps (blue) to keep the force measured on the red bead constant. (*c*) Force fluctuations *F*_*i*_ = *k* · *u*_*i*_ of two beads attached to opposite sides of a fibroblast cell.

Recently, we have introduced a novel method simultaneously to probe force generation and elastic response of cells using active and passive microrheology [16,17]. We here further develop this approach. Micrometre-sized polystyrene beads are attached to a rounded, suspended cell with a dual optical trap (figure 1*a*). Laser interferometry is then used to track the motion of the beads with high bandwidth (figure 1*b*). This approach allows us, on the one hand, to measure the force fluctuations generated by the cells. On the other hand, we can simultaneously probe the mechanical properties of the cell by applying oscillatory forces in the suspended geometry. In contrast to experiments with surface adherent cells of the widely used flattened geometry on substrates, we measure the total force transmitted to the outside world. Freely suspended cells have also been probed by the optical stretcher [18,19]. In that approach, cell-generated forces are not measurable, and cells are not attached to any matrix ligands, therefore not forming focal adhesions.

In rounded cells, actin and myosin are mainly confined to the less than 1 µm thick cellular cortex [20–22]. The round shape of suspended cells demonstrates surface tension competing with osmotic pressure [5,23,24]. Unlike lipid vesicles under osmotic pressures, cells store excess lipid membrane area in different types of surface invaginations [25,26]. Surface tension is thus generated by the acto-myosin cortex. Myosin has been shown to be able to contract and compact the actin networks [27,28]. Here, we demonstrate that the cortical acto-myosin network is the key cytoskeletal component generating contractile force fluctuations and providing mechanical strength, whereas the contribution of the microtubule network is negligible.

## 2. Results

### (a) Cell stiffness

**Figure 2.**
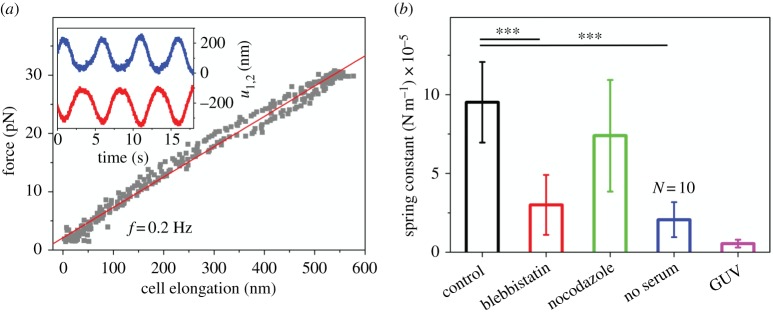
Active measurement of cell elasticity. (*a*) Force–elongation plot from a periodic stretching experiment, averaged over five periods. Inset: Displacement curves of bead 1 (red) and 2 (blue) in an active measurement. (*b*) Effective cell spring constants of fibroblast cells under control conditions (black), after treatment with 100 µM blebbistatin (red), 3 µM nocodazole (green) and in serum-free medium (blue). Effective spring constant of DOPC/DOPE-biotinyl giant lamellar vesicles (GUV) (purple). Ten cells measured for each condition (GUV, *N* = 6).

We first measured effective cell stiffness using the active method and applying oscillatory force, using the acousto-optic deflector to move one of the optical traps (figure 1*b*, electronic supplementary material, video S1). The inset in figure 2*a* shows an example of the time courses of displacements *u*_1_(*t*) and *u*_2_(*t*) of the two particles from the trap centres of the stationary and oscillating trap, respectively (defined in figure 1*b*). The driving amplitude of the AOD-steered trap *d* minus the difference between displacements *u*_1_ and *u*_2_ gives the effective elongation of the cell Δ*l* = *d* − (*u*_1_ − *u*_2_), and the applied force *F* = *k*(*u*_2_ − *u*_1_). Approximating the cell as a linear-elastic element and using Hooke's law:2.1
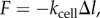
we then obtain an effective spring constant *k*_cell_ from force–elongation plots for each individual cell. Figure 2*a* shows an example of such a force–elongation relation for a 3T3 fibroblast in normal medium conditions (control). Averaging over 10 individual cells yields an effective spring constant of *k*_cell_ = 9.5 ± 2.5 × 10^−5^ N m^−1^ (figure 2*b*).

To distinguish the contributions of actin, myosin and microtubules to cellular compliance, we performed active deformation experiments after biochemical perturbations and determined the effective spring constant *k*_cell_ for each condition (figure 2*b*). Blocking the motor activity of non-muscle myosin II motors (NMM IIs) with 100 µM blebbistatin led to a threefold decrease in cell stiffness *k*_cell_ = 3.1 ± 1.5 × 10^−5^ N m^−1^ (figure 2*b*) compared to the cells in control conditions (*t*-test, significance level 99.9%). This result clearly confirms the important contribution of the cortical acto-myosin network to the mechanical rigidity and integrity of the cell. When deforming rounded cells, for which most of the volume does not contain filamentous actin, the more evenly distributed microtubule network might influence the overall mechanical response. Cells treated with 3 µM nocodazole, however, showed an only slightly smaller stiffness *k*_cell_ = 7.5 ± 4.1 × 10^−5^ N m^−1^ than untreated ones. This difference was not statistically significant. The microtubule network is thus not notably involved in determining cell compliance. However, it is possible that subtle changes were not detectable above the error margin of approximately 30%. A further possible perturbation of cellular contractility is serum-starvation. It is known that cells on substrates in low-serum or serum-free conditions show significantly reduced contractile forces [29,30]. Consistent with these findings, we found that cells actively probed 30 min after transfer to serum-free medium showed a significant reduction (*p* > 99.9%) in cell stiffness *k*_cell_ = 2.1 ± 1.2 × 10^−5^ N m^−1^, compared with the control population. This reduction in stiffness is comparable to the one seen after blebbistatin treatment. This is an indication that serum starvation is an efficient method to shut down cellular force fluctuations.

To estimate roughly the possible contribution of the plasma membrane to the elastic response of the cell, we also determined the effective spring constant of giant unilamellar vesicles (GUV). GUVs had a significantly (*p* > 99.9%) 30-fold lower effective spring constant than the control cells. One should note that this comparison merely provides an upper limit for the contribution due to bilayer bending. In an osmotically stretched GUV, uniaxial deformation would couple to both bending and stretching of the bilayer against osmotic pressure. Bilayer stretching is not relevant in cells owing to membrane stores. Since the resulting stiffness containing both contributions is much lower than that which we measure with cells, it is safe to conclude that the mechanical contributions from the lipid bilayer are negligible compared with the acto-myosin cortical network.

### (b) Contractile force fluctuations

In the passive mode, we used stationary optical traps and the interferometric detection system to monitor bead displacements caused by fluctuating forces generated by the cells. It is interesting to compare cell compliance with force fluctuations for the different biochemical conditions. The stationary optical traps were initially set to positions corresponding to approximately zero force (figure 1*c*). Since the cells are held suspended between the two trapped beads, the forces that the cells exert on the beads must be equal and opposite, *F*_1_ =−*F*_2_, with our definition of the axes (figure 1*b*). This is true (figure 1*c*) at least for longer timescales or low frequencies, where viscous drag in the medium is negligible. To analyse force fluctuations quantitatively, we calculated the Fourier transform of the cross-correlation of the two displacement time traces 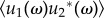. From this, we obtain the power spectral density (PSD) of the force fluctuations 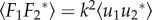 by multiplication with the trap stiffnesses, assumed to be equal here. Note that the amplitudes *u*_1_ and *u*_2_ do not have to be equal and opposite if the trap stiffnesses are not equal, but the forces have to be. Therefore, 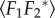 is a proper, always positive PSD. Consistent with earlier results [13,16], the frequency dependence of the PSD follows a scaling law 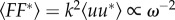 as shown in the electronic supplementary material, figure S1*.* This frequency dependence corresponds to a mean squared displacement scaling linearly with time. Although entirely unrelated, the same scaling form is found for simple thermal diffusion in a purely viscous environment. In our experiment, we know from the active compliance measurements that the beads are attached to a predominantly elastic cell where thermal fluctuations produce a cross-correlation that is almost flat in the frequency domain [31]. Therefore, the observed scaling must be caused by the non-equilibrium stress fluctuations generated by internal motor activity.

We checked our measurements for dependence on temperature and found no statistically significant differences between 23°C and 37°C (see the electronic supplementary material, figure S5).

As a quantitative estimate for total cellular force production, we integrated the force PSDs in the range from 0.1 to 10 Hz which captures the largest part of the motor-driven non-equilibrium stress fluctuations. By plotting total cellular force production versus trap stiffness, we confirmed the increase of transmitted forces with increasing trap stiffness (electronic supplementary material, figure S2) that was shown earlier and can be explained by a simple spring model [16] described in more detail in the electronic supplementary material. Briefly, the higher the trap stiffness, the more cell-generated force is transmitted to the traps. At trap stiffnesses higher than 2 × 10^−5^ N m^−1^, the total transmitted force leveled off which means that it approached the effective cellular stiffness, consistent with the results of the active measurements.

**Figure 3.**
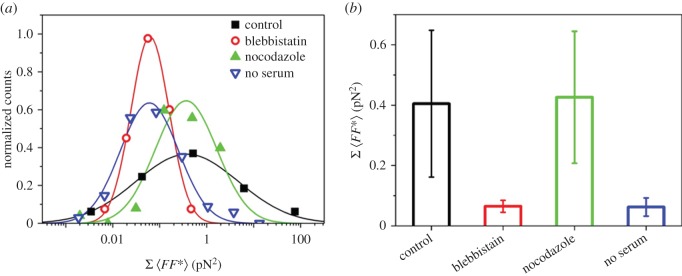
Cellular force fluctuations in the presence of cytoskeletal drugs. (*a*) Histograms of integrated force fluctuations (0.1–10 Hz) of control cells (black) and of cells after treatment with 100 µM blebbistatin (red), 3 µM nocodazole (green) and in serum-free medium (blue). (*b*) Integrated force fluctuations, averages and standard errors obtained from Gaussian fits of distributions showing that the distributions are broader for control and serum-starved cells in comparison to blebbistatin-treated and nocodazole-treated cells.

We then used experiments at trap stiffnesses higher than 2 × 10^−5^ N m^−1^ to compare the contributions of different key components of the cytoskeleton to the total force generation of cells by drug perturbation experiments. Figure 3*a* shows normalized histograms of integrated cellular force fluctuations, and figure 3*b* presents mean values of integrated force fluctuations. For untreated 3T3 fibroblasts (controls), we find a value of 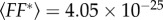 N². Blebbistatin treatment reduced force fluctuations by almost an order of magnitude, and the width of the distribution was a factor of 2.5 smaller than that of the control cells. In contrast to cells treated with blebbistatin, we saw no significant differences from the control cells when we treated the cells with 3 µM nocodazole, both in amplitude and width of distribution. Force fluctuations of serum-starved cells, in contrast, were strongly diminished in comparison to the control cells and were comparable to those of blebbistatin-treated cells. In comparison with the blebbistatin-treated cells, integrated force fluctuations were distributed twice as broadly in serum-free medium. It appears therefore that the reaction of cells to serum starvation is more variable from cell to cell than the direct inhibition of NMM II by blebbistatin. Overall, our results suggest a major role of the acto-myosin network in producing the cellular force fluctuations that does not depend on the integrity of the microtubule cytoskeleton.

### (c) Force-clamp experiments

In all experiments described so far, the force acting on the cell through the optical traps was initially adjusted to zero, but typically slowly fluctuated substantially away from zero when the cells changed their shape with time. Since cells are expected to sense external forces and react to them, it should be informative to observe cells under defined constant forces. For this purpose, we constructed a force clamp using feedback to keep the externally applied force constant at a defined value. We repositioned one of the two optical traps to compensate for changes in force measured by the other trap due to slow cell-shape variations. We then measured cellular force fluctuations at different fixed values of pre-tension. Electronic supplementary material, figure S3*a*, shows the displacement fluctuations of the two beads attached to a cell while we alternated the set value of pre-tension between 5 and 25 pN, roughly every 2 min. There was an immediate elastic response of the cell to the changing force, but after that no stereotypic further reaction. Rather, the cells performed slow irregular fluctuations on the scale of minutes that appeared fairly independent of the imposed force jumps and total clamp force. Such slow oscillations have also been found by others [32]. We also observed similar irregular fluctuations during long-time observations without a force clamp (see the electronic supplementary material, figure S4). Electronic supplementary material, figure S3*b*, shows power spectral densities of the cell diameter fluctuations under the three different force-clamp conditions that approximately follow the same scaling law 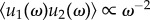 seen without force clamp and discussed in §2b. There was no significant difference for different clamp forces (5, 25 and 50 pN). This implies that the cell is creating strain fluctuations more or less independently of the external pre-stress. This result implies that the forces generated by the cell driving its own deformation must be much larger than the maximal forces generated by the traps. Note that this is not a contradiction to the result that the trap stiffness was found to be comparable to the cell stiffness.

**Figure 4.**
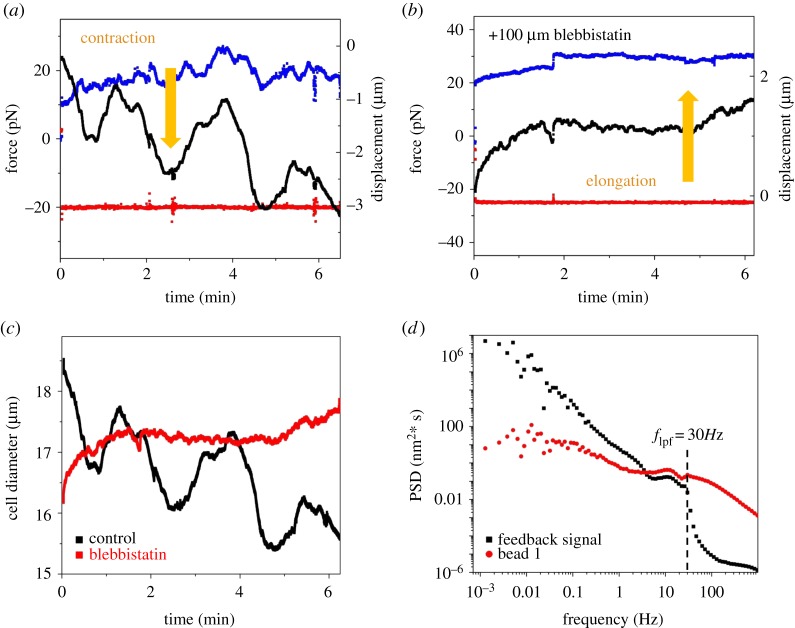
Cell deformations under force-clamp conditions. (*a*) Constant force measurement at 20 pN. Force (left *y*-axis) on bead 1 (red squares) is clamped, bead 2 is moved by AOD, force on bead 2 (blue squares) is measured with errors due to trap movement, black squares show feedback-controlled position of trap 2 (right axis). Over time we observe a periodically contracting cell (decreasing and negative numbers of displacement) with increasing amplitude. (*b*) Constant force measurement at 25 pN of a blebbistatin-treated cell. Feedback signal shows a slowly elongating cell (larger and positive values of displacement). (*c*) Cell diameter fluctuated and decreased due to contractile forces under external force clamp (black), and the blebbistatin-treated cell showed relaxation behaviour and elongation (red). (*d*) Power spectral density of the feedback signal and of the displacement of the bead in the stationary trap with a feedback low-pass filter at *f*_lpf_ = 30 Hz. Below the filter frequency, all cell movements were followed by the feedback while the relative position of the bead with respect to the trap centre remained constant. Above the filter frequency, the feedback does not follow the fluctuations.

To test whether cells react to an externally imposed constant force on a longer timescale, we observed cells in a force-clamp experiment for more than 6 min. Figure 4*a* shows force fluctuations and cell-shape changes of a cell in culture medium at a clamped force of 20 pN. Cells showed overall irregular contractions, possibly slow oscillations. To check whether these deformations were actively driven by the actin/myosin cortex, we repeated the experiment in the presence of 100 µM blebbistatin (figure 4*b*). Blocking the NNM II motors strictly prevented any contraction. Furthermore, cells treated with blebbistatin systematically elongated under the imposed load by more than 1.5 µm (for the cell shown in figure 4*b*) after 6 min as expected for a viscoelastic material. In figure 4*c*, cell diameter is plotted versus time for both experiments, showing the contracting and expanding control cell and the slowly relaxing blebbistatin-treated cell, both under a force clamp.

Figure 4*d* shows the residual fluctuation spectrum of the stationary bead under force-feedback conditions and the error signal controlling the moving trap. The feedback incorporates a 30 Hz low-pass filter. Below the filter frequency, the bead fluctuations are at a constant noise-determined level, and the error signal reports the cellular contractions against its internal stiffness. This spectrum also shows the *ω*^−2^ power-law scaling as expected. Above the filter frequency, the bead is recording the cellular force fluctuations against the now stationary trap.

## 3. Discussion

Cells in tissue are mechanically connected to the extracellular matrix and neighbouring cells. Forces transmitted via these connections tie into regulatory networks and allow the cells to probe and feel their environment. Our experiments show that the essential player determining the mechanics of suspended rounded-up cells is the cortical acto-myosin network, which provides mechanical stability against internal osmotic pressure and also drives shape fluctuations. Control experiments showed that the effective spring constant of an osmotically weakly stretched GUV of similar size as the cells is four times lower than even that of blebbistatin-treated and serum-free cells. This shows that, even in this worst-case scenario of a completely stretched geometry, the lipid envelope of the cell provides no major contribution to the overall elasticity of a fibroblast cell. The measured effective spring constant of the cells *k*_cell_ = 9.5 ± 2.5 × 10^−5^ N m^−1^ needs to be related to material properties taking into account geometry and the strong volume constraint imposed by osmotic pressure. In a naive estimate, assuming the cell to be an elastic solid, the spring constant can be related to an effective Young's modulus *E* of approximately 100 Pa [16]. This is comparable to the values obtained by atomic force microscopy (AFM; making similarly naive assumptions) and optical trapping for small deformations [33]. One should keep in mind that this is not a material constant but rather a descriptive parameter dependent on both geometry and material constants of the cellular components.

The fact that the depolymerization of microtubules with nocodazole had no significant effect on cell stiffness is consistent with the generally accepted wisdom that actin dominates the elastic response of most adherent cells [33,34]. Although Brangwynne *et al.* [35] showed that microtubules embedded in an elastic matrix can bear orders of magnitude higher compressional forces than bare microtubules, this effect seems not to be relevant in our experimental geometry. Inhibiting NMM II motors by blebbistatin led in our experiments to an about threefold decrease in the effective cellular stiffness. This shows that cortical tension generated by NMM II can strongly modulate actin-based elastic response. While it is well known that myosin I also contributes to cortical tension in cells [36], blebbistatin is highly specific for myosin II [37] and therefore does not affect myosin I. Future experiments will allow dissection of the distinct contributions of different myosin motors to cortical tension and force production. Stiffening in response t internal stress is, indeed, a characteristic property of semiflexible polymer networks and has been seen in experiments with model networks before [13,38]. Interestingly, blebbistatin-treated cells suspended in an optical stretcher appear to stiffen (Jochen Guck 2014, personal communication), possibly pointing to nonlinear effects or to a role of integrin-mediated adhesion domains for the contractile acto-myosin cortex.

Stress fluctuations also indicate the molecular origin of cortical tension. The effect of blebbistatin implicates myosin as the responsible molecular motor. The *ω*^−2^ power law in the force PSD is a signature expected at shorter times than the characteristic attachment time of the motors [38]. Minifilaments of NMM are likely to produce holding times of the order of 10 s, and *ω*^−2^ scaling behaviour has also been observed in experiments with probe particles embedded in cells [10,39] and in cytoskeletal model networks [13]. These reports of characteristic myosin force production times of the order of 10 s are one order of magnitude shorter than the observed oscillation periods. We therefore think that more complex and collective effects of cellular force generation and transduction cause the observed slow shape fluctuations.

The temperature independence of the amplitude of force fluctuations is in contrast to experiments with cells adhering to a two-dimensional substrate, which were shown by AFM to generate higher stresses at higher temperatures [40]. Different behaviour is not unexpected, though, since the active processes driving cell spreading are clearly different from those creating fluctuations of the rounded-up cells. The lower amplitude force fluctuations in serum-free medium are consistent with a report that serum is necessary to fully phosphorylate the myosin II regulatory light chain [41].

## 4. Conclusions

Mechanical probing of suspended fibroblasts in a dual optical trap has shown that both total cellular force generation and effective cell elasticity of spherical 3T3 fibroblasts are dominated by the acto-myosin cortical network. Biochemical perturbation with nocodazole showed that the microtubule network does not significantly contribute to either. We demonstrate that manipulation of cells in suspended three-dimensional geometry provides an alternative window on cell mechanics, complementary to common methods probing adherent cells and amenable to quantitative modelling.

## 5. Material and methods

### (a) 3T3 Fibroblasts

3T3 fibroblast cells (#ACC 173, Leibniz-Institut DSMZ, Braunschweig, Germany) were cultured in Dulbecco's modified Eagle's medium (DMEM) (D6046, Sigma-Aldrich, St. Louis, MO, USA) with 10% fetal bovine serum (FBS, #F0244, Sigma-Aldrich) and 1% penicillin–streptomycin (#17-602E, Lonza, Basel, Switzerland), at 37°C and 5% CO_2_. Cells were passaged in culture flasks using 0.05% trypsin (#59417C, Sigma-Aldrich) every 2–3 days and seeded in new flasks at a density of roughly 125 000 cells per flask (75 cm^2^, #83.1813, Sarstedt AG, Nümbrecht, Germany).

For experiments, cells grown to approximately 80% confluence were trypsinized for 3 min at 37°C and centrifuged after adding DMEM with FBS. The pellet was resuspended in CO_2_-independent medium (#18045–054, Life Technologies, Darmstadt, Germany) also supplemented with 10% FBS and stored on ice until use within 8 h.

For the drug perturbation experiments, trypsinized cells were incubated in a test tube with either 100 µM blebbistatin (racemic mixture) (#203389, Merck, Darmstadt, Germany) or 3 µM nocodazole (M1404, Sigma-Aldrich) for 30 min at room temperature prior to the experiment.

To prevent surface attachment of the cells in our sample chamber, glass coverslips were silanized with dimethyldichlorosilane (DDS, #85126, Sigma) and incubated for 5 min with 1% pluronic F108 (#3402.13, BASF, Ludwigshafen, Germany).

A total of 4 µm diameter carboxylated polystyrene beads (PPs-4.0COOH, Kisker Biotech, Steinfurt, Germany) were coated with fibronectin (or biotin–neutravidin for vesicle experiments) (#F0895, #40945, Sigma-Aldrich; for details, see the electronic supplementary material. For measurements, 100 µl of the cell stock solution, 50 µl of the pluronic solution and 5 µl of the bead solution were added to 900 µl CO_2_-independent medium.

### (b) Vesicle preparation

Giant unilamelar vesicles (GUVs) were grown from a mixture containing 95% of 1,2-dioleoyl-*sn*-glycero-3-phosphocholine (DOPC) and 5% 1,2-dioleoyl-*sn*-glycero-3-phosphoethanolamine-*N*-(cap biotinyl) (DOPE-B-Cap) (#850375, #870273, Avanti Polar Lipids, Alabaster, AL, USA) by electroswelling in a solution of 100 mM glucose (#X997.2, Carl Roth, Karlsruhe, Germany) and 1 mM NaCl (#1064041000, Merck) as described in [42], and stored at 4°C for up to one week.

### (c) Dual optical trap

Our custom-built dual optical trap is similar to the one previously described [17]. Deviating from the earlier work, the two optical traps were created by splitting the polarized 1064 nm laser into two beams with polarizing beam splitters as described in [43,44]. Since both traps originate from one laser, we made sure that laser beam-pointing fluctuations did not move the two traps in opposing directions which would cause anti-correlated signals obscuring our measurements. Slow parallel beam motions do not affect the force measurements between the two beads. For constant-force measurements, a force feedback was implemented acting on one of the traps, realized using a custom LabView program (National Instruments, Austin, TX, USA) using the built-in PID-algorithm (see the electronic supplementary material) fed with the position fluctuation signal of the other (stationary) trap. To move one trap, an acousto-optical deflector (AOD, DTD 276HB6, IntraAction, Corp., Bellwood, IL, USA) was driven by a voltage-controlled oscillator (VCO, AA.DRF. 40, AA Sa, Orsay, France). The location of this laser focus was controlled in such a way that the displacement signal (proportional to force) from the stationary trap was maintained at a constant preset value.

### (d) Passive and active measurements of cell mechanical properties

The dual optical trap was first used to capture two beads and attach them on opposite sides of a rounded-up cell (figure 1*a*). Each fibronectin-coated bead was allowed to bind to the cell for approximately 5 min to form a tight adhesion domain. The position fluctuations of the beads were then recorded by quadrant photo diodes (QPDs) placed in a plane conjugate to the back-focal plane of the condenser lens of the microscope (figure 1*b*) [45]. Voltage signals from the QPDs were acquired and digitized using a field programmable gate array (FPGA) and LabView software (NI PXI-7833R, National Instruments). Microrheology measurements were performed as described previously [16,17]. In brief: for passive measurements, we recorded the position fluctuations of the two beads *u*_*i*_(*t*) (figure 1*c*) and calculated the Fourier transform of the cross-correlation of the bead positions 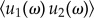 by custom MATLAB programs (MathWorks, Natick, MA, USA) as described previously [17]. Multiplying position cross-correlation with trap stiffness *k* converts to force cross-correlation. Trap stiffness was determined by fitting the PSD of the Brownian motion of each bead in the sample solution before attaching it to the cell [46]. To obtain an estimate of the total force production of the cells, we integrated the force spectrum over the range 0.1–10 Hz to get 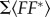 (see the electronic supplementary material, figure S1).

For the active compliance measurements, the AOD was used to oscillate spatially one optical trap while keeping the other one at a constant position (electronic supplementary material, video S1). Displacements of approximately 500 nm amplitude with a frequency of 0.2 Hz were applied, which remains within the linear-elastic response regime of the cell [16]. Positions of both beads and the centre of the oscillating trap were recorded and the force applied to the cell and its elongation were calculated. All experiments were carried out at room temperature (23°C) unless stated otherwise.

## Supplementary Material

Supplementary Information and Figures
