# Supplementary material for: Force fluctuations in three-dimensional suspended fibroblasts
Source: Philos Trans R Soc Lond B Biol Sci. 2015 Feb 5;370(1661):20140028. doi: 10.1098/rstb.2014.0028 (PMC4275901; doi:10.1098/rstb.2014.0028)
Supplement: Supplementary Information and Figures [file rstb20140028supp1.pdf]

Force fluctuations in 3D suspended fibroblasts

Florian Schlosser, Florian Rehfeldt *, and Christoph F. Schmidt *

Third Institute of Physics – Biophysics, Georg August University, 37077 Göttingen, Germany

*corresponding authors (FR) rehfeldt@physik3.gwdg.de and (CFS) cfs@physik3.gwdg.de

Phil. Trans. R. Soc. B. **370** doi: 10.1098/rstb.2014.0028

Supplementary Information

Force Feedback

To be able to maintain cells under a constant tension, we added a self-written force feedback mode to our optical trap setup. We used the PID.vi included in the LabView PID palette.

The QPD signal of the optical trap that is not steered with the AOD is acquired using a freely programmable gate array (FPGA) which is then read out by a LabView program for data processing. The signal is filtered with a software low pass filter and fed into a PI algorithm as the process variable. The low pass filtering assures that the PI algorithm only follows the low frequency force fluctuations of the cell. The feedback algorithm calculates an error signal and an output value according to the setpoint force. The output value is then fed back to the FPGA analog output. A voltage divider and impedance converter is used to adapt current and voltage of the output signal to the voltage controlled oscillator (VCO) which steers the AOD in the trapping laser beam path.

Bead coating

For fibronectin coating, 1 ml of a 50 mg/ml solution of 4µm polystyrene beads is diluted in 10 ml water and washed two times in MilliQ (EMD Millipore Corporation, Billerica, MA, USA) water. After second wash, the pellet is resuspended in 10 ml PBS and transferred into a beaker. While mixing the bead solution, 100 mg of N-(3-Dimethylaminopropyl)-N'-ethylcarbodiimide hydrochloride (EDC, E6383, Sigma) is added. After 15 min of reaction at room temperature, the solution is washed two times in MilliQ and resuspended after the second wash in 5 ml PBS. 350 ml of fibronectin are added and the solution is stirred at room temperature for 2-4 hours. After this reaction, the solution is washed again, resuspended in 4 ml of quenching solution (0.15 g glycine/50 ml MilliQ + 1 ml of 10% BSA in PBS, A1377, AppliChem GmbH, Darmstadt, Germany and A9418, Sigma) and gently mixed for 30 minutes at room temperature. The solution is washed again and resuspended in storage buffer (0.1% of BSA in PBS) at desired concentration and stored at 4°C until usage.

Spring Model

Our measurement setup is able to record cellular force fluctuations by analyzing the position of the beads within their respective optical traps. It is worthwhile noting that this can be understood within the framework of a simple spring model, where the cell is represented by an effective spring constant k_{cell}, that is suspended between two springs (the beads in the traps) of spring constant k as described in (Mizuno et al. Ref in main text). This model leads to the following relation of the total cellular force fluctuations $\langle FF^* \rangle$ with the spring constants k_{cell}, k and the measured bead displacements $\langle uu^* \rangle$.

$$k^2 \sum \langle uu^* \rangle = \left[\frac{k^2}{(k + 2k_{cell})^2} \right] \sum \langle FF^* \rangle$$

For high trap stiffnesses, transmitted force fluctuations saturate. We fitted the data taken at different trap stiffnesses with two free parameters for the effective cell stiffness k_{cell} and the total cellular force fluctuations $\langle FF^* \rangle$ as shown in Fig. S2. The resulting value for the effective spring constant in serum-free conditions $k_{cell} = 1.9 \cdot 10^{-5}$ Nm^{-1}, is consistent with the results from the active compliance measurements and the total cellular force production is estimated to be $\langle FF^* \rangle = 4.04 \times 10^{-25}$ N^2.

Supplementary Figures

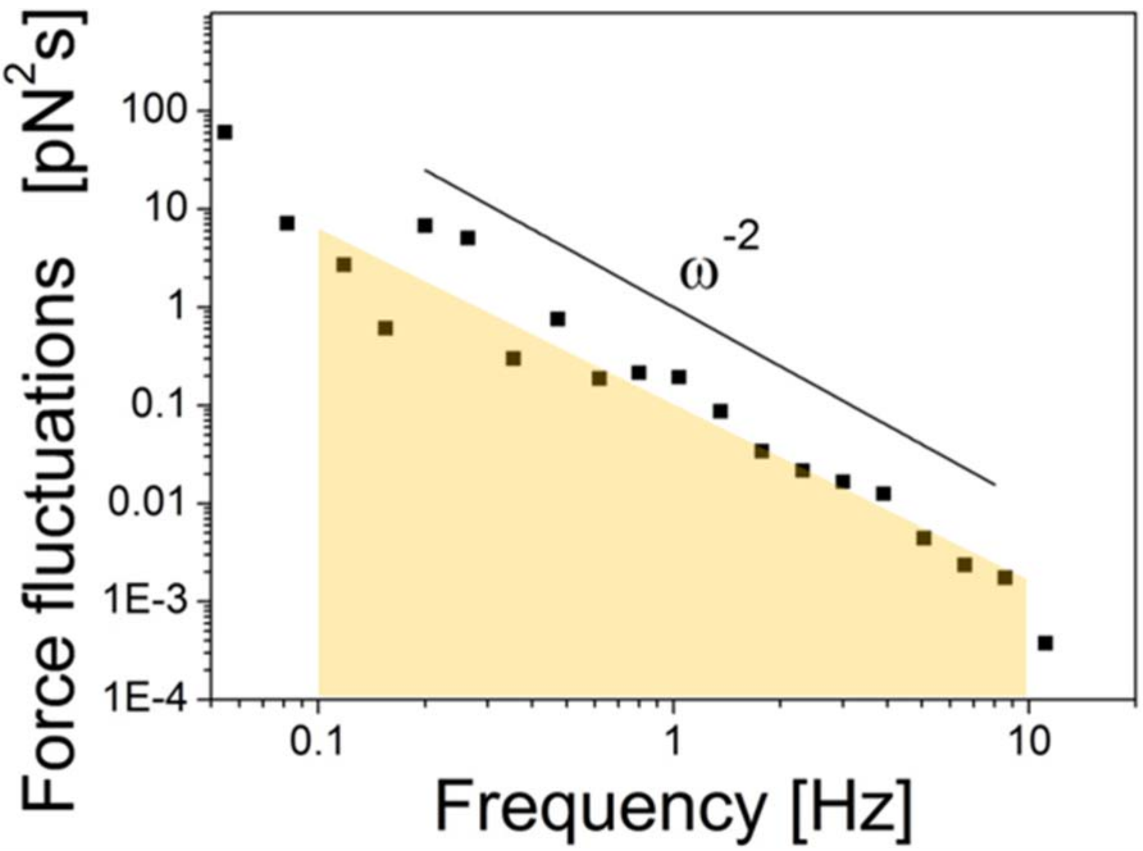

Figure S1: Force spectrum of a fibroblast cell. The orange box marks the integration window for each measurement to get the sum of force fluctuations $k^2 \sum u_1(\omega) u_2(\omega)$. The solid black line represents a ω^{-2} power law.

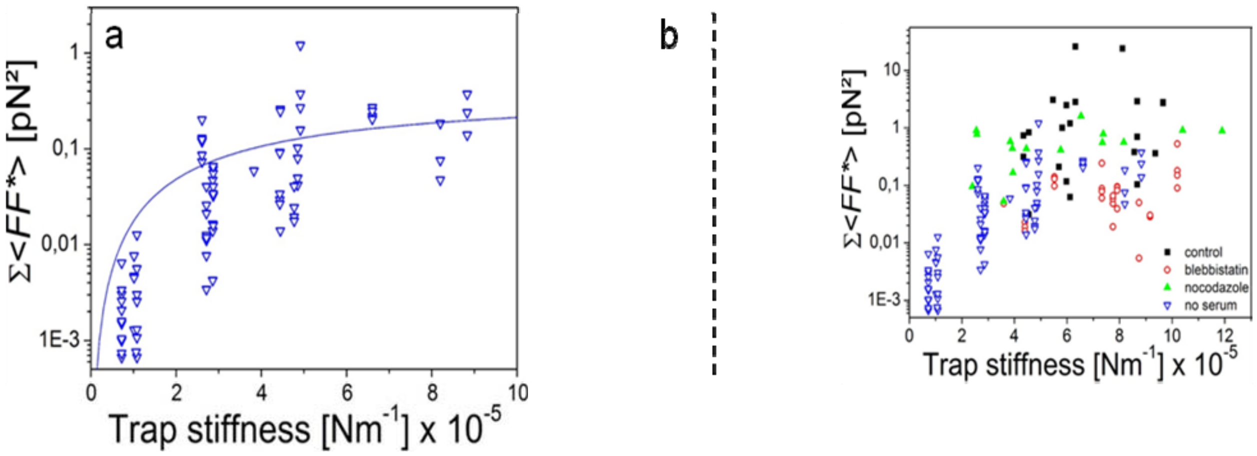

Figure S2: (a) Force fluctuations of fibroblast cells in serum-free medium trapped at different trap stiffness. Each point shows force fluctuations of an individual cell obtained by integrating the force spectrum in the range from 0.1 to 10 Hz (fig. S1). The fit shows the spring model from (Mizuno et al. 2009) **(b)** Force fluctuations of fibroblast cells trapped at different trap stiffness and treated with different biochemical agents. For the force histograms only data points at a trapping stiffness higher than 2x10^{-5} N/m were chosen, where the cellular force fluctuations show roughly a plateau.

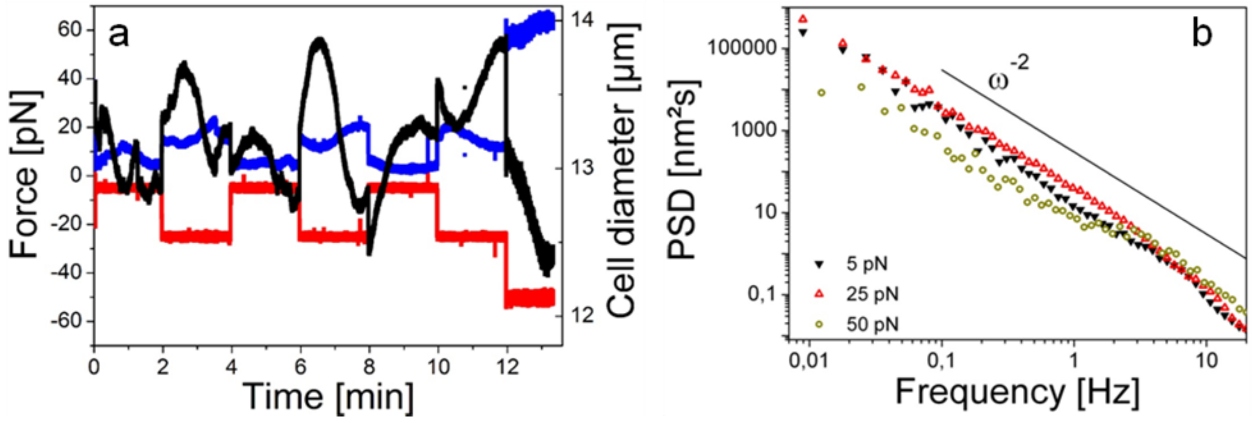

Figure S3: (a) Constant force measurement, force switching between 5 and 25 pN. Red and blue data points show applied force on bead 1 and bead 2 (left axis), black signal shows trap position (right axis). Higher values show contracting cell. **(b)** Powerspectral density of the trap fluctuation signal.

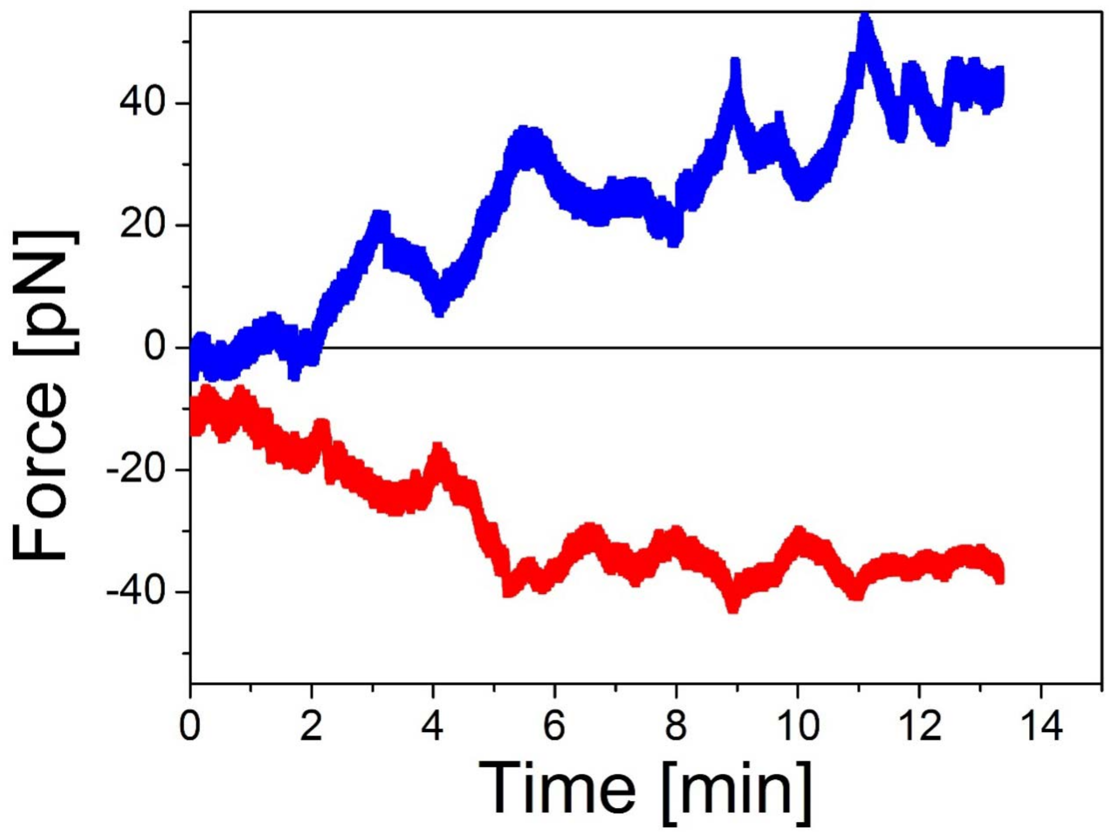

Figure S4: Long term force fluctuations of two beads attached to opposite sides of a fibroblast cell. Short time periodic force fluctuations on top of an overall contraction of the cell at the timescale of the whole experiment are evident.

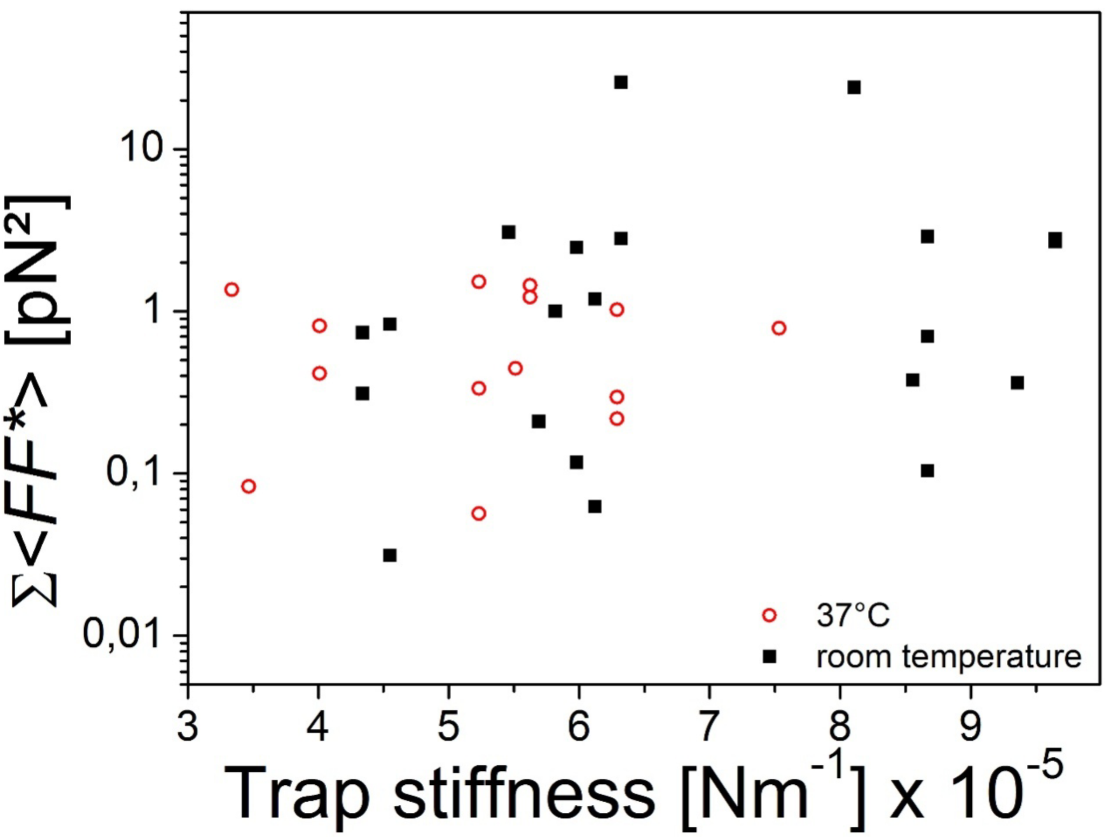

Figure S5: Force fluctuations of fibroblast cells at different trap stiffness. Black data shows cells at room temperature, open red data shows cells at 37°C. This data shows no significant difference for the force fluctuations at room temperature and at 37°C.

[Supplied separately]

Video S1: Video of a periodic stretching experiment. Two beads are attached to a fibroblast cell and held in position with optical traps. One trap (right) is oscillated by an AOD. Stretching amplitude is 680 nm.
